# Metastatic Uterine Adenosarcoma Presenting as Haemobilia and a Rare Cause of Gastrointestinal Haemorrhage: A Case Report and Review of the Literature

**DOI:** 10.7759/cureus.86849

**Published:** 2025-06-27

**Authors:** Mauro Sousa, Pedro Rodrigues, Catarina Pato, Teresa Braga, Daniel Jordão, José P Feire, Luís Miranda

**Affiliations:** 1 General Surgery, Unidade Local de Saúde de Santa Maria, Lisbon, PRT

**Keywords:** case report, gallbladder metastasis, haemobilia, metastatic uterine adenosarcoma, upper gastrointestinal bleeding

## Abstract

Haemobilia, defined as bleeding into the biliary tract, is a rare but significant cause of upper gastrointestinal haemorrhage. While its aetiology commonly includes trauma, vascular malformations, and primary hepatobiliary malignancies, haemobilia secondary to metastatic disease is exceedingly rare. Uterine adenosarcomas are uncommon mesenchymal malignancies, and, to our knowledge, metastasis to the gallbladder has not been previously reported.

This case report describes a 53-year-old female with a history of uterine adenosarcoma, who presented with haemobilia caused by metastatic disease. Initial symptoms included abdominal pain and melena, with imaging revealing gallbladder wall thickening and small bowel intussusception. The patient underwent urgent laparoscopic cholecystectomy and subsequently required further surgeries for recurrent bowel obstructions due to metastatic lesions. Histopathological examination confirmed metastatic uterine adenosarcoma in the gallbladder and small bowel. This rare case highlights the challenges in diagnosing and managing metastatic haemobilia, emphasizing the importance of considering metastatic disease in patients with gastrointestinal bleeding and a history of uterine sarcoma. A multidisciplinary approach, including surgery and palliative chemotherapy, proved crucial for treatment. This case underscores the need for awareness regarding such rare presentations to ensure timely intervention and improve patient outcomes.

## Introduction

Haemobilia, defined as bleeding into the biliary tract, is an uncommon yet significant cause of upper gastrointestinal haemorrhage [1-4]. This phenomenon was first described in 1654 by Francis Glisson in Cambridge, describing bleeding into the biliary tree. However, it was Sandblom in 1948 who used the term “haemobilia” in his paper [2]. Its aetiology encompasses a range of conditions, including trauma, iatrogenic interventions, vascular malformations, and neoplasms [2-5]. Among neoplastic causes, primary hepatobiliary tumours are more frequently implicated; metastatic lesions leading to haemobilia are exceedingly rare [3-8]. 

Uterine sarcomas represent a heterogeneous group of malignancies arising from the mesenchymal tissues of the uterus, accounting for approximately 3%-7% of all uterine cancers [9-12]. Adenosarcomas, a subtype characterized by a combination of benign epithelial and malignant stromal components, represent about 8% of uterine sarcomas [10,11,13]. These tumours typically present as polypoid masses within the endometrial cavity and are most commonly diagnosed in postmenopausal women, although cases in younger patients have been documented [11]. Clinical manifestations often include abnormal uterine bleeding, pelvic pain, or the presence of a pelvic mass [9]. 

The metastatic behaviour of uterine adenosarcomas varies, with common sites including the lungs, liver, and peritoneal surfaces [14,15]. Metastasis to the gallbladder is exceptionally rare, with limited cases reported in the literature [16,17]. The propensity for vascular invasion, particularly lymphovascular emboli, has been associated with an increased risk of metastasis and poorer prognosis in uterine adenosarcomas [18,19]. 

Haemobilia resulting from metastatic uterine adenosarcoma is an extraordinarily uncommon clinical scenario. The pathophysiological mechanism likely involves tumour invasion into the biliary vasculature, leading to haemorrhage within the biliary tree [3-5,16]. Clinically, patients may present with the classic Quincke’s triad: right upper quadrant pain, jaundice, and gastrointestinal bleeding, though this triad is observed in a minority of cases [2,3,5]. Diagnosis is often challenging and requires a high index of suspicion, with imaging studies such as contrast-enhanced computed tomography (CT) or magnetic resonance imaging (MRI) playing a crucial role [2-5]. Endoscopy and angiography may further aid in both diagnosis and therapeutic intervention [20,21]. 

Management of haemobilia involves stabilizing the patient, controlling the bleeding source, and addressing the underlying aetiology. In cases secondary to metastatic disease, treatment options may include endoscopic or angiographic haemostasis, surgical resection if feasible, and systemic therapy tailored to the primary malignancy [3-5,22]. 

To our knowledge, this is the first reported case of haemobilia caused by metastatic uterine adenosarcoma, highlighting the diagnostic and therapeutic complexity of this presentation.

This report presents a rare case of haemobilia secondary to metastatic uterine adenosarcoma, contributing to the limited literature on this unusual manifestation. It underscores the importance of considering metastatic disease in the differential diagnosis of haemobilia - particularly in patients with a history of uterine sarcoma - and highlights the challenges in the diagnosis and management of such complex cases. 

## Case presentation

**Figure 1:**
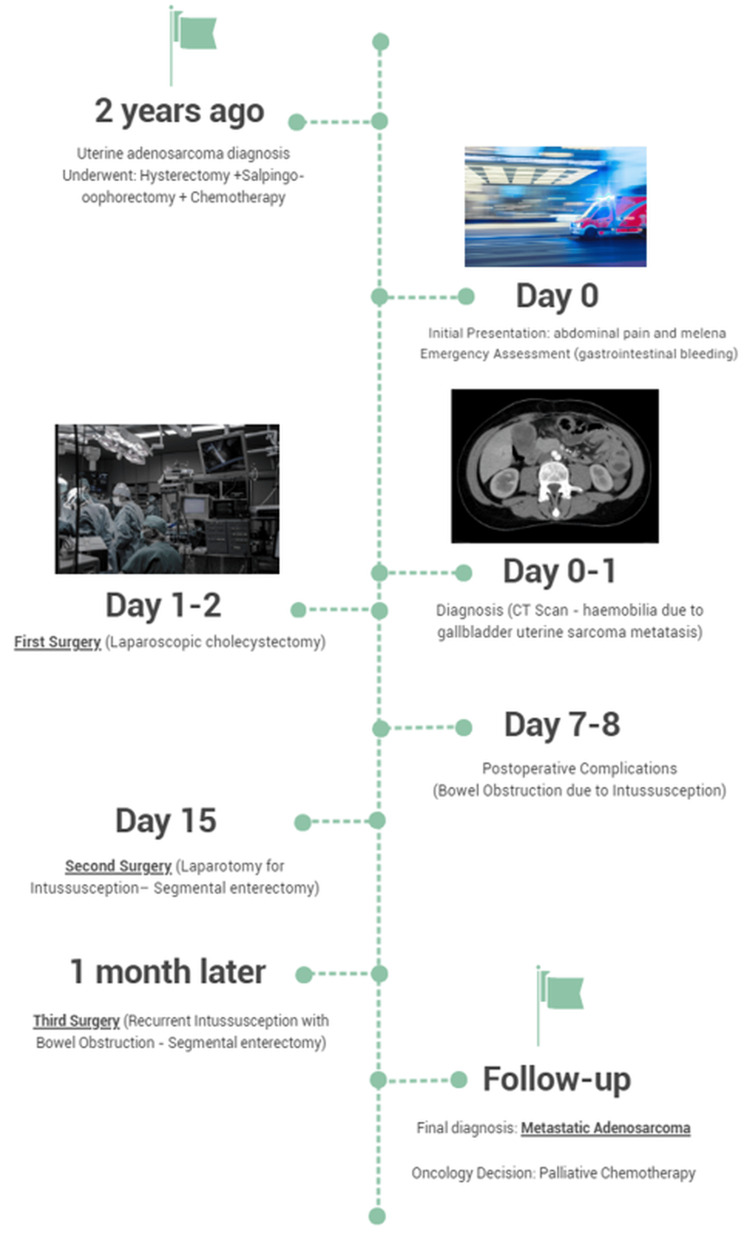
Chronological overview of clinical progression and treatments. CT, computed tomography

This clinical case concerns a 53-year-old female patient who presented to the Emergency Department with complaints of general malaise, weakness, dizziness, fatigue, abdominal pain, and dark stools lasting one day (Figure 1). She denied fever, nausea, and vomiting.

The patient was originally from Angola but had been residing in Portugal for many years. She had a relevant medical history of uterine adenosarcoma with FIGO (International Federation of Gynecology and Obstetrics) Stage IB (localized tumour, ≥50% myometrial invasion), endometrioid carcinoma treated one year and five months prior. The treatment included total hysterectomy, bilateral salpingo-oophorectomy, adjuvant chemotherapy for the adenosarcoma, and brachytherapy for the endometrioid carcinoma. She was under Gynaecological and Oncology follow-up for suspected pulmonary metastasis, undergoing evaluation for potential surgical treatment (lobectomy). Additionally, she was a heterozygous carrier for sickle cell disease and had a recent history of iron-deficiency anaemia, managed by immunohaemotherapy. Over the previous six months, she had a baseline haemoglobin level that fluctuated around 6.2 g/dL, requiring recurrent red blood cell transfusions and intravenous iron. Her family history was notable for uterine fibroids. She was not on any regular home medications; however, she had received intermittent intravenous iron therapy under the supervision of the Immunohematology Department due to recurrent anaemia.

Upon admission to the Emergency Department, the patient was haemodynamically stable, with a blood pressure of 116/76 mmHg and heart rate of 93 bpm. She was eupneic on room air, with a normal respiratory rate, and had a Glasgow Coma Scale (GCS) score of 15, indicating full alertness and orientation. Abdominal examination revealed generalized tenderness, more pronounced in the right hypochondrium. Digital rectal examination revealed traces of dark stools consistent with melena. Gynaecological examination was unremarkable, with no vaginal bleeding, lesions, or observable collections. 

Laboratory tests revealed a haemoglobin level of 8.5 g/dL, an increase from 7.9 g/dL measured three days earlier following one unit of packed red blood cells, with no abnormalities in coagulation parameters, inflammatory markers, or liver function tests. Coagulation studies were within normal limits, including an International Normalized Ratio (INR) of 1.1 and a platelet count of 527,000/µL.

**Figure 2:**
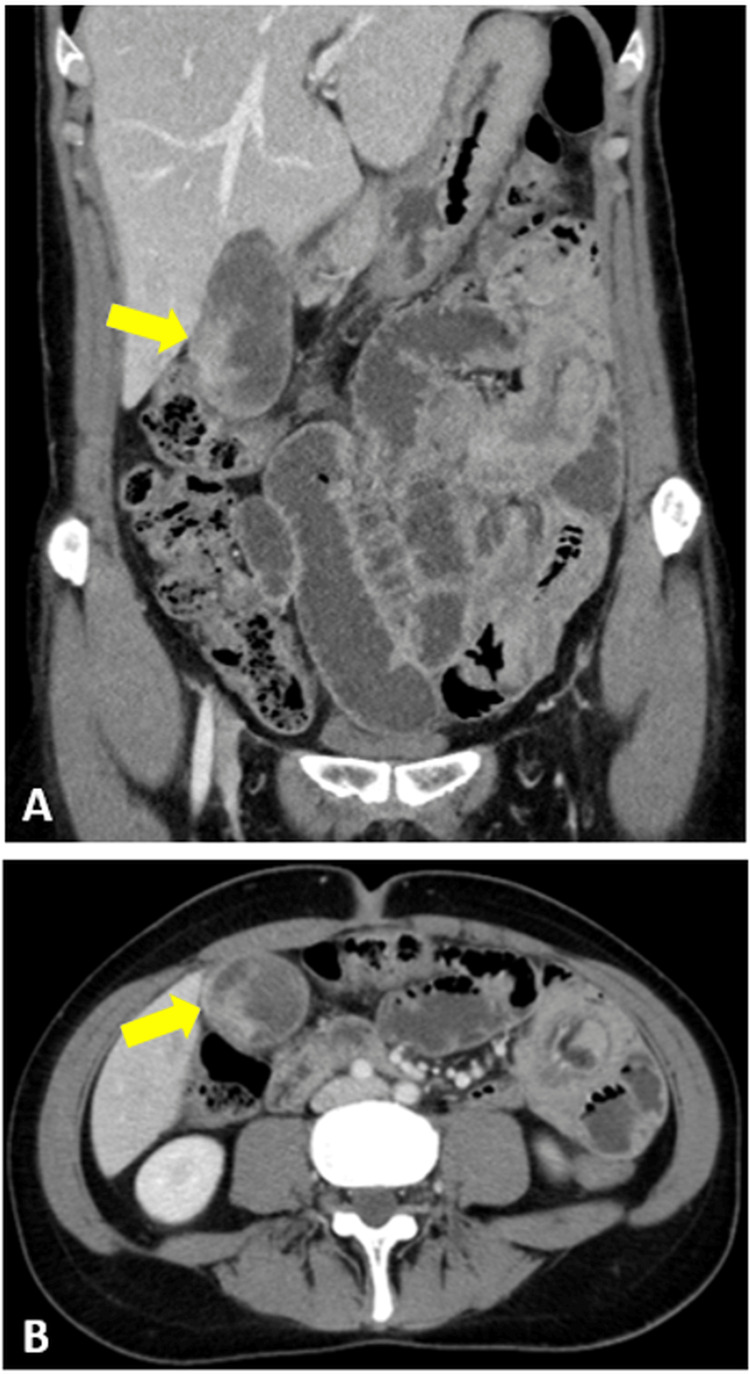
CT images on the day of Emergency Department admission demonstrating gallbladder haemorrhage (indicated by a yellow arrow). (A) Coronal plane; (B) Axial plane CT, computed tomography

Due to the presence of melena and suboptimal transfusion response, an abdominopelvic CT scan was performed, raising suspicion of gastrointestinal bleeding. The CT scan revealed a gallbladder with thickened and irregular walls - findings suggestive of intraluminal haemorrhage, likely secondary to uterine adenosarcoma metastasis (Figure 2). Mild dilation of the intrahepatic bile ducts and the main bile duct, without a clear obstructive cause, was also noted. Furthermore, three segments of small bowel intussusception were identified, with wall thickening in the involved segments but no upstream dilation. 

Based on the clinical and imaging findings, the patient was evaluated by the General Surgery team and diagnosed with haemobilia due to gallbladder haemorrhage, likely secondary to metastatic uterine adenosarcoma. An urgent surgical intervention was indicated, and the patient underwent exploratory laparoscopy and laparoscopic cholecystectomy.

**Figure 3:**
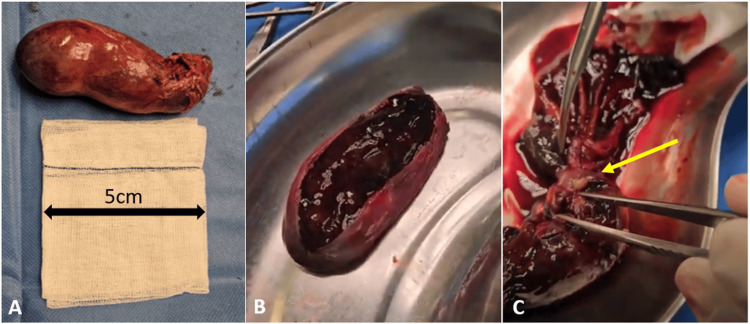
Gross appearance of the gallbladder specimen, after laparoscopic cholecystectomy (operative day 0). (A) External surface with congested and thickened wall; (B) Longitudinal section showing intraluminal haemorrhage and dark blood clots; (C) Mucosal surface with irregularities and macroscopic features suggestive of haemorrhagic-tumoral infiltration (arrow).

Given the patient’s haemodynamic stability, absence of clinical signs of bowel obstruction, and imaging findings predominantly suggestive of gallbladder haemorrhage without clear evidence of obstructive pathology, a laparoscopic approach was initially chosen. During surgery, a laparoscopic cholecystectomy was performed along with systematic exploration of the abdominal cavity, including inspection of the small bowel (Figure 3). No signs of intussusception or bowel dilation indicative of intestinal obstruction were identified at that time. Additionally, there was no evidence of peritoneal seeding or intra-abdominal adhesions. The procedure was uneventful.

In the immediate postoperative period, the patient showed good clinical progress during the first week, with no evidence of further bleeding and normalization of stool. However, by the end of the first week, she developed symptoms of nausea and vomiting. A follow-up CT scan revealed three segments of small bowel intussusception with upstream dilation. Conservative management was initially attempted, but persistent symptoms of bowel obstruction and rising inflammatory markers led to surgical intervention. 

On the 15th postoperative day, the patient underwent urgent reoperation. Initial exploratory laparoscopy revealed limited intra-abdominal space and technical difficulties, prompting conversion to a mini median laparotomy. Two lesions were identified: a 30-cm ileal intussusception and a 10-cm jejunal retraction causing stenosis. Segmental enterectomies were performed with primary anastomosis. The postoperative course was favourable, with gradual reintroduction of oral diet and normalization of bowel function, and the patient was discharged on the 20th day after the second surgery. 

One month after the second surgery, the patient returned to the Emergency Department with symptoms suggestive of bowel obstruction, including nausea and vomiting. A CT scan revealed a proximal small bowel intussusception as the obstructive point. She underwent urgent surgery via mini median laparotomy. Two proximal jejunal lesions were identified: one causing intussusception and the other causing retraction, both leading to bowel obstruction. A segmental enterectomy encompassing both lesions was performed with primary anastomosis. Once again, the postoperative course was favourable, with progressive oral intake and normalization of bowel transit, and the patient was discharged on the 21st day following surgery. 

Histopathological examination of all excised lesions (gallbladder and intestinal segments) confirmed metastatic uterine adenosarcoma with high-grade features, including over 30 mitoses per high-power field, and a proliferative index of 70%. 

The case was discussed in a multidisciplinary tumour board involving Oncology, Gynaecology, General Surgery, Radiology, Radiation Therapy, and Anatomical Pathology. Considering the context of multiple organ metastases, palliative chemotherapy was recommended. The patient is currently under oncology follow-up with stable disease.

## Discussion

The occurrence of haemobilia as a result of metastatic uterine adenosarcoma, as demonstrated in our case, highlights a rare but significant complication of this condition. Although it has been reported that several types of cancer can metastasize to the gallbladder, such as adenocarcinoma of the stomach, extrahepatic bile duct, colon, and rectum; mucinous adenocarcinoma of the appendix; melanoma; renal cell carcinoma; hepatocellular carcinoma; and uterine cervix squamous cell carcinoma [16,17], the pathophysiology in our case likely involves direct invasion of the biliary vasculature by metastatic tumour deposits, causing haemorrhage within the biliary tree and presenting as upper gastrointestinal bleeding [3-5,22]. 

Symptoms are rare in patients with metastatic tumours of the gallbladder. When present, acute cholecystitis is the most common presentation, often followed by jaundice due to common bile duct obstruction. While this is, to the best of our knowledge, the first reported case of haemobilia secondary to metastatic uterine adenosarcoma, clinicians managing cancer patients with signs or symptoms of cholecystitis should consider the possibility of gallbladder metastases. 

CT, MRI, and angiography aid in diagnosing and treating hypervascular lesions. Management focuses on stabilization, bleeding control, and cause-specific interventions, prioritizing minimally invasive methods [2-5,20,21]. 

To prevent symptoms or complications, cholecystectomy is indicated for patients with a symptomatic gallbladder, regardless of whether the tumour is primary or metastatic. In asymptomatic cases, cholecystectomy may also be considered to improve survival in patients with isolated disease [7,8,16,17].

The prognosis for gallbladder metastasis is poor, reflecting the advanced stage of the malignancy and the presence of lymphovascular invasion. Early recognition and prompt management remain essential to improve clinical outcomes [14,15,18,19,23].

## Conclusions

Clinicians should maintain a high index of suspicion for haemobilia in patients presenting with gastrointestinal bleeding and a history of uterine sarcoma. A comprehensive diagnostic approach, coupled with timely therapeutic interventions, is crucial in managing this complex condition.
